# An overview of cancer/testis antigens expression in classical Hodgkin's lymphoma (cHL) identifies MAGE-A family and MAGE-C1 as the most frequently expressed antigens in a set of Brazilian cHL patients

**DOI:** 10.1186/1471-2407-11-416

**Published:** 2011-09-28

**Authors:** Riguel J Inaoka, Achim A Jungbluth, Otávio CG Baiocchi, Mariane CG Assis, Nicole C Hanson, Denise Frosina, Jodie Tassello, Adriana B Bortoluzzo, Antonio C Alves, Gisele WB Colleoni

**Affiliations:** 1 Departamento de Oncologia Clinica e Experimental, Universidade Federal de Sao Paulo. Rua Botucatu, 740, Vila Clementino, Sao Paulo, SP 04023-900, Brazil; 2 Ludwig Institute for Cancer Research New York Branch, Memorial Sloan-Kettering Cancer Center, 1275 York Avenue, Box 32, New York, NY 10021-6007, USA; 3 Instituto de Ensino e Pesquisa, Insper. Rua Quata, 300, Vila Olimpia, Sao Paulo, SP 04546-042, Brazil; 4 Departamento de Anatomia Patologica, Universidade Federal de Sao Paulo. Rua Botucatu, 740, Vila Clementino, Sao Paulo, SP 04023-900, Brazil

**Keywords:** Hodgkin's Lymphoma, cancer/testis antigens

## Abstract

**Background:**

Cancer/testis antigens are considered potential targets for immunotherapy due to their tumor-associated expression pattern. Although recent studies have demonstrated high expression of CT45 in classical Hodgkin's lymphomas (cHL), less is known about the expression pattern of other families of CTAs in cHL. We aim to evaluate the expression of MAGE-A family, MAGE-C1/CT7, MAGE-C2/CT10, NY-ESO1 and GAGE family in cHL and to correlate their expression with clinical and prognostic factors in cHL.

**Methods:**

Tissue microarray was generated from 38 cHL archival cases from Pathology Department of Universidade Federal de Sao Paulo. Immunohistochemistry (IHC) was done using the following panel of antibodies: MAGE-A family (MA454, M3H67, 57B and 6C1), GAGE (#26), NY-ESO-1 (E978), MAGE-C1/CT7 (CT7-33) and MAGE-C2/CT10 (CT10#5).

**Results:**

We found CTA expression in 21.1% of our cHL series. Among the tested CTAs, only MAGE-A family 7/38 (18.4%) and MAGE-C1/CT7 5/38 (13.2%) were positive in our cHL samples. We found higher CTA positivity in advanced stage (28.6%) compared to early stage (11.8%) disease, but this difference was not statistically significant. Analysis of other clinicopathological subgroups of cHL including histological subtypes, EBV status and response to treatment also did not demonstrate statistical significant differences in CTA expression.

**Conclusion:**

We found CTA expression in 21.1% of cHL samples using our panel. Our preliminary findings suggest that from all CTAs included in this study, MAGE-A family and MAGE-C1/CT7 are the most interesting ones to be explored in further studies.

## Background

Classical Hodgkin lymphoma (cHL) is characterized by the presence of rare neoplastic Hodgkin-Reed-Sternberg (HRS) cells embedded in an inflammatory background of nonmalignant cells [1,2]. The mechanisms of how HRS cells survive in this inflammatory milieu remain controversial and the identification of specific antigens restricted to the HRS cells is crucial for the development of new treatment strategies, augmenting host antitumor immunological response.

Cancer/testis antigens (CTAs) are considered potential candidates for antigen-specific cancer immunotherapy due to their particular characteristics of high immunogenicity with no or highly restricted expression in normal tissues (testis and placenta) [3]. There are more than 100 CTA genes reported in the literature to date [4] but biological function of most CTAs remains poorly understood. Recent studies have provided some evidence that CTAs may have antiapoptotic properties rather than regulating cell proliferation or adhesion in cancer [5-12] and it could explain the persistence of minimal residual disease in some malignancies, even in cHL, where the potential of cure is very high. The frequency of CTA expression is highly variable among different tumor types. Melanoma, ovarian cancer, and lung cancer are considered tumors with high frequency of CTA expression, while hematopoietic malignancies, renal, colon and pancreatic cancers, are considered tumors with low frequency of CTA expression [3]. Some exceptions to this observation among hematopoietic malignancies are the high expression of CT7/MAGE-C1 in multiple myeloma [13,14], and CT45 in classical Hodgkin lymphoma (cHL) [15,16]. Studies correlating CTA expression with clinicopathological features in different tumor types have demonstrated the association of CTA positivity with higher tumor grade, advanced stage or metastatic disease and worse clinical outcome [13,17-25].

Considering that the available information about CTA expression in cHL is scarce and heterogeneous regarding methods and samples, we investigated the immunohistochemical expression against a broad panel of CTAs in cHL tissue samples to evaluate their potential as prognostic markers and candidates for immunotherapeutic approach in cHL patients.

## Methods

We retrospectively reviewed all cases of cHL diagnosed between 2004 and 2008 at the University Hospital São Paulo. Medical records from 38 adult patients (> 18 years) with cHL were reviewed and information on sex, age at diagnosis, Ann Arbor clinical stage, laboratory results, treatment used and response were retrieved. The response to primary treatment was classified according to the International Workshop criteria [26]. Patients treated with radiotherapy alone as first-line therapy and with positive HIV serology were excluded from this study. For this study, only cHL patients with tumors whose histology could be confirmed by hemopathologist re-review and paraffin blocks with enough material for tissue microarray (TMA) construction could be retrieved were studied. Sufficient data was obtained from 38 patients. All patients received ABVD (doxorubicin, bleomycin, vinblastine and dacarbazine) chemotherapy protocol and had locally extensive or advanced stage disease at diagnosis. Locally extensive disease was defined by clinical stages I-II-A/B (Ann Arbor Staging System) with massive mediastinal adenopathy (mass > 1/3 maximum intrathoracic diameter on standing postero-anterior chest x-rays), and advanced disease defined as stages III-IV. Determination of EBV-association in tumor biopsies was done by immunohistochemistry for latent membrane protein 1 (LMP1) following previously established procedures [27]. Samples from a known EBV-related cHL served as a positive control in each run.

This research was approved by the Ethical Review Committee of our Institution according to the Declaration of Helsinki (0998/07), and all patients provided written informed consent.

Formalin-fixed paraffin embedded tissues of 38 cHL were obtained from the archives of the Department of Pathology, Hospital São Paulo, UNIFESP, Brazil. All HL cases were classified according to WHO classification for hematologic malignancies [28] as cHL. 25 (65.8%) cases were classified as nodular sclerosis subtype, 9 (23.7%) as mixed cellularity, 2 (5.3%) as lymphocyte predominant and 2 (5.3%) were unclassified. Table 1 summarizes the clinical data of these patients. Slides from all cases were reviewed and representative blocks were chosen for tissue microarray.

**Table 1:** Clinical data of Hodgkin's lymphoma patients (n = 38).

Clinical data	n	%
**Median age: **28.5 (16-57)		
**Gender**		
Male	18	47.4
Female	20	52.6
**HL Subtype**		
Nodular sclerosis	25	65.8
Mixed cellularity	9	23.7
Lymphocyte predominant	2	5.3
Unclassified	2	5.3
**Ann Arbor staging**		
Early (I or II)	17	44.7
Advanced (III or IV)	21	55.3
**EBV status**		
Negative	21	55.3
Positive	17	44.7
**Response**		
Complete response	30	78.9
Relapsed/refractory	8	21.1

Two core-needle biopsies (1.0 mm diameter) from tumor representative areas of each HL case were obtained and then re-embedded in an array master block using techniques originally developed by Kononen *et al*. [29] and then modified by Hedvat *et al*. [30] and Beecher Instruments (Sun Prairie, WI, USA) arraying device.

The mAbs used for immunohistochemistry are listed in Table 2 [22,31-38]. The evaluation of MAGE-A family expression was done using a "MAGE-A cocktail" containing the following antibodies: MA454, M3H67, 57B and 6C1, that allowed an overview of MAGE-A family (MAGE-A1, MAGE-A2, MAGE-A3, MAGE-A4, MAGE-A6 MAGE-A10 and MAGE-A12) expression in HL.

**Table 2:** Monoclonal antibodies used in immunohistochemical analyses.

Monoclonal antibody	Mainly recognized Antigen(s)	Source	Cellular staining pattern	References
MA454*	MAGE-A1	LICR	Cytoplasmic	[22,31-34]
M3H67*	MAGE-A3	LICR	Nuclear & cytoplasmic	[22,33,34]
57B *	MAGE-A4	Dr Spagnoli, Basel, Switzerland	Nuclear & cytoplasmic	[22,33,34]
6C1*	MAGE-A 1, 2, 3, 4, 6, 10 and 12	LICR	Nuclear & cytoplasmic	[35]
**CT7-33**	CT7/MAGE-C1	LICR	Nuclear & cytoplasmic	[22,33,34]
**CT10#5**	CT10/MAGE-C2	LICR	Nuclear	[35-37]
**#26**	GAGE	BD Bioscience	Nuclear & cytoplasmic	[35,36]
**E978**	NY-ESO-1	LICR	Cytoplasmic	[36,38]

*MAbs included in "MAGE-A cocktail" used to search MAGE-A family expression in HL TMA.

Testis with preserved spermatogenesis was used as positive controls and reactive lymph nodes and tonsils samples were used as negative controls for all antibodies.

Associations between the variables were tested by the Pearson Chi-Square Test (X^2^). Mann-Whitney test was used to perform mean comparisons. A p value lower than 0.05 was considered as statistically significant.

## Results

The majority of patients presented advanced disease and B symptoms at diagnosis. We found a strong correlation between anti-LMP1 identification on HRS cells and response to ABVD regimen, with all 21 EBV-negative patients achieving complete response (CR) and only 9/17 (52.9%) of EBV-positive patients achieving CR (p < 0.001). None of other clinical subgroups presented statistical significant difference in treatment response, as demonstrated on Table 3.

**Table 3:** Hodgkin's lymphoma treatment response analysis.

Clinical data	N	Complete response (%)	N	Refractory/ relapsed (%)	p value
**Gender**					
Male	13	72.2	5	27.8	0.286
Female	17	85.0	3	15.0	
**HL Subtype**					
Nodular sclerosis	21	84.0	4	16.0	0.220
Mixed cellularity	5	55.6	4	44.4	
Lymphocyte predominant	2	100.0	0	0	
Unclassified	2	100.0	0	0	
**Ann Arbor staging**					
Early (I or II)	15	88.2	2	11.8	0.195
Advanced (III or IV)	15	71.4	6	28.6	
**EBV status**					
Negative	21	100.0	0	0	**< 0.001**
Positive	9	52.9	8	47.1	
**CTA positivity**					
0 CT	24	80.0	6	20.0	0.954
> or = 1	6	75.0	2	25.0	
**MAGE-A**					
negative	24	77.4	7	22.6	0.538
positive	6	85.7	1	14.3	
**CT7**					
negative	27	81.8	6	18.2	0.279
positive	3	60	2	40	

**Figure 1:**
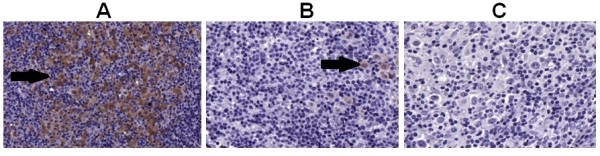
**Immunohistochemical staining of three different nodular sclerosis cHL samples using an anti-CT7 antibody (CT7-33)**. (A) Diffuse positivity. (B) Focal positivity. (C) Negative staining for CT7-33; ×400.

Due to the low overall CTA expression in cHL TMA, positivity was not graduated, and the samples were analyzed as positive (any degree of positivity) or negative. Figure 1 demonstrates three nodular sclerosis cHL samples stained for CT7. Sample (A) shows diffuse positivity, sample (B) is focally positive and sample (C) is a negative case.

Eight (21.1%) of 38 cHL cases expressed at least one CTA, being 6/25 nodular sclerosis and 2/9 mixed cellularity. Among the 8 positive cases, 6/20 were female and 2/18 male. Despite the difference found in CTA expression between early (11.8%) and advanced stage (28.6%) disease, this difference was not statistically significant (p = 0.195). None of the other clinical subgroups of cHL according to histological subtypes, EBV status and response to treatment also presented statistically significant difference in CTA expression, as demonstrated on Table 4. Among the tested CTAs, only MAGE-A family 7/38 (18.4%) and MAGE-C1/CT7 5/38 (13.2%) were positive in our cHL samples. CT10/MAGE-C2, GAGE and NY-ESO-1 were negative in all samples. Four of these samples were positive for both CTAs.

**Table 4:** Hodgkin's lymphoma CTA expression analysis.

Clinical data	N	0 CTA (%)	N	> or = 1 CTA (%)	p value
**HL Subtype**					
Nodular sclerosis	19	76.0	6	24.0	0.752
Mixed cellularity	7	77.8	2	22.2	
Lymphocyte predominant	2	100.0	0	0	
Unclassified	2	100.0	0	0	
**Ann Arbor staging**					
Early (I or II)	15	88.2	2	11.8	0.195
Advanced (III or IV)	15	71.4	6	28.6	
**EBV status**					
Negative	17	81.0	4	19.0	0.522
Positive	13	76.5	4	23.5	
**Treatment Response**					
Complete response	24	80.0	6	20.0	0.954
Non Complete response	6	75.0	2	25.0	

## Discussion and conclusions

In our study, the majority of cases had B symptoms (68.4%) and advanced disease (65.7%) at diagnosis, as seen in other developing countries. The positivity for EBV was 44.7% in our cohort of cHL. According to literature, the incidence of EBV-related cHL is highly variable among different populations and higher EBV positivity has seen in underdevelopment countries compared to developed ones. In previous studies, Oliveira *et al*. and Vassallo *et al*. found EBV positivity in 52.5% and 64.1% of Brazilian cHL patients respectively [39,40]. The prognostic significance of EBV expression is controversial, but the correlation of EBV positivity and poorer outcome has been described in some studies [41]. The difference of treatment failure rate between EBV positive (47.1%) and EBV negative patients (0%) was statistically significant (p < 0.001) in our cohort.

Using our panel, we found expression of CTAs in 21.1% of cHL patients. MAGE-A family was positive in 18.4% and MAGE-C1/CT7 in 13.2% of samples. Despite the availability of few studies evaluating the expression of CTAs in cHL to date, our findings are in agreement with most of studies that evaluated the expression of same CTAs in cHL using RT-PCR or immunohistochemical analysis. Expression of MAGE-A family in cHL was evaluated by Chambost *et al*. [42] and it was positive in 28% of cHL samples using RT-PCR and in 21% cHL tissue sections using immunohistochemistry with an anti-MAGE-A4 antibody. Recently, Chen *et al*. [15] described the expression of CT45 in cHL and other B-cell lymphomas. They found expression of CT45 in 58% of cHL by immunohistochemistry. A previous study published by Heidebrecht *et al*. [16] also described the expression of CT45 in 55% of pediatric and adolescent cHL using immunohistochemistry, reinforcing CT45 as one of the most promising CTA to be explored in cancer vaccine trials for cHL to date. Unfortunately, CT45 could not be included in our immunohistochemistry panel because specific antibodies were not commercially available, neither at Ludwig Institute for Cancer Research - NY, our collaborating center at the time of this study. Another CTA family whose expression pattern was studied in cHL was SSX. Colleoni *et al*. [43] found positivity of SSX family in 15.6% of 32 cHL samples. In summary, except for CT45, the available studies suggest an overall positivity for other CTA families tested in cHL of about 10% to 30%. We found a tendency to a higher CTA expression among advanced stage disease compared to early stage disease in our cHL samples as described in literature [13,17-25], but the difference did not reach statistical significance.

Therefore, our results emphasize the importance of studying CTAs expression in a Brazilian set of patients. We acknowledge the fact that our small sample size may have influenced these results, and larger studies are warranted. However, our results point toward a relationship between CTA expression and disease severity.

Further studies investigating CTA expression on HRS cells and how they can elicit humoral and cytotoxic response will contribute not only to our understanding on the pathogenesis of cHL but also to the development of new CTA-directed immunotherapy, particularly for advanced stage cases and for patients who did not achieve a complete response after initial therapy.

## Competing interests

The authors declare that they have no competing interests.

## Authors' contributions

This manuscript is a product of a PhD research project (RJI) conducted under the supervision of GWBC. AAJ, NH, DF and JT (Ludwig Institute for Cancer Research - NY) participated in the decision of CTAs to be included in our study, provided the anti-CTA antibodies and carried out the immunohistochemistry. OCGB and MCGA participated in acquisition, analysis and interpretation of cHL clinical data. ABB performed the statistical analysis. ACA reviewed all cHL tumor sample slides. RJI participated in all steps described above and drafted the manuscript. GWBC have been involved in conception and design of this study, analysis, interpretation of data and final approval of the version to be published. All authors read and approved the final manuscript.

## Pre-publication history

The pre-publication history for this paper can be accessed here:

http://www.biomedcentral.com/1471-2407/11/416/prepub
